# Publisher’s Note

**DOI:** 10.1093/oons/kvad002

**Published:** 2023-03-24

In regards to:

Harold A Burgess, Edward A Burton, A critical review of zebrafish neurological disease models–1. The premise: neuroanatomical, cellular, and genetic homology, and experimental tractability, *Oxford Open Neuroscience*, 2023;, kvac018, https://doi.org/10.1093/oons/kvac018

*and*

Edward A Burton, Harold A Burgess, A critical review of zebrafish neurological disease models–2. Application: functional and neuroanatomical phenotyping strategies and chemical screens, *Oxford Open Neuroscience*, 2022;, kvac019, https://doi.org/10.1093/oons/kvac019,

The publisher apologizes to readers for any confusion caused by the advance access publication dates for these articles. Together, these articles comprise a two-part review and should have been published with the same advance access publication date. For the avoidance of doubt, below are the relevant manuscript processing dates for both articles.

https://doi.org/10.1093/oons/kvac018

Received: October 2, 2022

Revised: November 13, 2022

Accepted: December 1, 2022

Advance access publication: January 6, 2023

Corrected proof publication: March 3, 2023

https://doi.org/10.1093/oons/kvac019

Received: October 2, 2022

Revised: November 28, 2022

Accepted: December 1, 2022

Advance access publication: December 9, 2022

Corrected proof publication: March 3, 2023

These details have been reinforced in this Publisher’s Note to preserve the published version of record.

